# Peer review of "High-Modulus Laminated SiC/AZ91 Material with Adjustable Microstructure and Mechanical Properties Based on the Adjustment of the Densities of the Ceramic Layers"

_materials, 2023, doi:10.3390/ma16186168_

Round 1

Reviewer 1 Report

1.     Authors must put references in support of the claims made in the manuscript. In the introduction section in lines 27, 28, and 29 “However, 27 the application of magnesium alloys in these fields is limited by inherent drawbacks, such 28 as low absolute strength and modulus of elasticity.” This statement should be supported by some references. There are many places in the manuscript where references need to be added. Authors are advised to carefully go through the whole manuscript once.

2.     The motive for doing the research is not well stated in the last paragraph of the introduction. Please rewrite the same.

3.     In section 2.2, the authors stated various parameters for sample preparation. Are these values novel in nature? if yes how many iterations were performed to reach these optimum parameters, if not what about the previous references published for these values?

Author Response

Dear reviewers,

Thank you for your letter and for the reviewers’ comments concerning our manuscript entitled “High Modulus Laminated SiCp/AZ91 Material With Adjustable Microstructure and Mechanical Properties by Adjusting the Densities of the Ceramic Layers” (ID: materials-2559464). Those comments are all valuable and very helpful for revising and improving our paper, as well as the important guiding significance to our researches. We have studied comments carefully and have made correction which we hope meet with approval. Below summarizes the details of the answers to the questions, and revisions made in the manuscript, in which the original comments are written in plain black text, the responses in blue text, and the changes in the manuscript are in red text. We hope that the amendments and responses make the revised manuscript better readable and satisfied with the editor and reviewers. If not, we really appreciate to have the chance to reconsider your additional comments in future communication.

Reviewers' comments:

1.Authors must put references in support of the claims made in the manuscript. In the introduction section in lines 27, 28, and 29 “However, 27 the application of magnesium alloys in these fields is limited by inherent drawbacks, such 28 as low absolute strength and modulus of elasticity.” This statement should be supported by some references. There are many places in the manuscript where references need to be added. Authors are advised to carefully go through the whole manuscript once.

Response 1: Much thanks for the reviewer’s valuable advice! We have carefully checked the whole manuscript and added references to support the corresponding claims. Some examples are listed below:

  • Line 28-30 Page1: However, the application of magnesium alloys in these fields is limited by inherent drawbacks, such as low absolute strength and modulus of elasticity [3].
  • Line 43-44 Page1-2: Among these methods, the freeze casting technique provides an effective means of achieving preform conformational modulation [10].
  • Line 50-54 Page2: For instance, in the case of SiC preforms, high temperature sintering involves the reaction of SiC to produce SiO2, which then reacts with a sintering aid to form a low melting point aluminum silicate. This silicate fills the pores within the SiC particles and facilitates their connection [11].
  • Line 79-80 Page2: This demonstrates the clear relationship among sintering temperature, looseness within the ceramic layer, and material forming [18].
  • Line 181-183 Page5: Furthermore, a diffuse distribution of Al, Y, and O elements near the particles suggests dispersion of the sintering aids surrounding the SiC particles, serving as bonding agents [21].
  • Line 197-199 Page5: Mullite phase generated melts to form a liquid phase, which fills the interstices be-tween ceramic particles, resulting in enhanced bonding among them and increased bond strength [23].
  • Line 206-208 Page6: Due to the favorable wettability of SiO2 and Mg, the Mg component reacts with the residual SiO2 during the infiltration process, resulting in the formation of Mg2Si and Mg2SiO4 [25].
  • Line 363-364 Page10: These findings indicate that the ceramic layer and the metal layer undergo coordinated deformation during loading [38].
  • Line 418-419 Page11: As cracks in various regions continue to propagate and penetrate the ceramic layer, the interface acts as a barrier to further crack propagation [46].

  1. The motive for doing the research is not well stated in the last paragraph of the introduction. Please rewrite the same.

Response 2: Much thanks for the reviewer’s valuable advice! We have rewritten the last paragraph of the introduction. The relevant changes are as follows:

Therefore, the primary purpose of this study is to explore the preparation of magnesium matrix composites with high strength and toughness based on the sintering temperature control of the looseness in the ceramic layer. In this work, the layered SiC preforms were prepared by freeze casting and sintered at 800°C, 900°C and 1000°C, respectively. Subsequently, the AZ91 magnesium alloy was hydraulically pressed into the pores and interlayer gaps of the SiC preform layer under pressure to prepare the SiCp/AZ9 magnesium base material. On this basis, the effects of sintering temperature on the internal structure, microstructure, mechanical properties and fracture behavior of the laminated SiCp/AZ91 Material were studied.

  1. In section 2.2, the authors stated various parameters for sample preparation. Are these values novel in nature? if yes how many iterations were performed to reach these optimum parameters, if not what about the previous references published for these values?

Response 3: Much thanks for the reviewer’s valuable advice! These parameters of sample preparation are innovative. The specific process parameters in the sample preparation process are explored as follows.

  • Line 109-112 Page3: Firstly, in order to improve the strength of the preform after sintering, the preforms with sintering aids content of 0%, 3%, 6% and 10% were prepared respectively. As shown in Supplementary1, it was found that when sintering aids content was 10%, the compressive strength exceeds 25MPa, which meets the subsequent preparation conditions. Secondly, the dispersant is to ensure that all particles in the slurry are evenly dispersed to avoid sedimentation during the freezing process. The preforms with its content of 0.3%, 0.4% and 0.5% were prepared respectively. It was found that when the dispersant content was 0.4%, the slurry was well dispersed and no sedimentation occurred during the preparation process. In order to facilitate the subsequent elimination of dispersants and reduce the impact on the composition of SiC preforms, the dispersant content was selected to be 0.4%.

Supplementary Fig. 1 Compressive strength of SiC preforms with different sintering aids content (a) 0% (b) 3% (c) 6% (d) 10%

  • Line 115-117 Page3: Freezing temperature is one of the most important parameters in the freeze casting method, and its change will significantly affect the microstructure of the preform. The SiC preforms prepared at different freezing temperatures are shown in Supplementary Fig.2.Although their configurations are different, they are all layered structures. This work is to explore the effect of sintering temperature on the microstructure and properties of composites, and the effect of sintering temperature on the density of SiC skeleton. Therefore, this manuscript fixed -30°C as the freezing temperature to prepare SiC preforms, and then sintered at different temperatures to control the density by sintering temperature, so as to study the related properties of the composites. In addition, the effect of freezing temperature on the microstructure and properties of composite materials will be elaborated in subsequent research.

Supplementary Fig. 2 Microstructure of SiC porous preforms with an initial solid content of 30 vol. % at different freezing temperatures. (a) -10℃; (b) -20℃; (c) -30 ℃; (d) -50 ℃;

Reviewer 2 Report

The article by Zeqi Du et al. presents a study of the influence of sintering temperature on the microstructure, mechanical properties and fracture behavior of composites with a magnesium matrix SiCp/AZ91. The introduction substantiates the relevance of the research topic, presents methods for increasing the strength and impact resistance of composites with a ceramic matrix. The article is written in a good scientific language, links to current sources of literature are given. I think that the article will be interesting to readers and can be published in this form

Author Response

Thank you very much for your affirmation of this manuscript!

Reviewer 3 Report

This paper is easy to read, and the logic is clear. But the authors fail to highlight their data in the abstract, and some parts of the main content raised my technical concerns. I recommend a major revision.

1.       In the abstract, the authors mention “toughness” with no data support – They should add their toughness number here.

2.       When it comes to the Mg properties in abstract, they should be specific about the exact numbers for those properties: Exactly how light? How high the specific strength is? Etc.

3.       The processing involves freeze casting, sintering, and infiltration, which are confusing by pure words (in introduction and methods part). The authors should add a process/procedure illustration plot to show the critical process parameters.

4.       “crack initiation in composites is primarily attributed to the ceramic layer”: I have doubt for this claim. Indeed, it is the interface of ceramics and metals in composites that contribute to the crack initiation, as there is lattice mismatch. Please learn from Ref. [1-2] and have a more solid discussion.

[1] Geng, J., Li, Y., Xiao, H., Li, H., Sun, H., Chen, D., Wang, M. and Wang, H., 2021. Study fatigue crack initiation in TiB2/Al-Cu-Mg composite by in-situ SEM and X-ray microtomography. International Journal of Fatigue, 142, p.105976.

[2] Nanoparticles enabled mechanism for hot cracking elimination in aluminum alloys. M Sokoluk, J Yuan, S Pan, X Li. Metallurgical and Materials Transactions A 52 (7), 3083-3096

5.       You used Al2O3 and Y2O3 as sintering aid. How do you confirm they are gone after sintering? If they are still there (as told in your Figure 2), can they react with Mg? Why? Need more explanation.

6.       Equation 1-5: Need to add the Gibbs free energy change (I think this will also help my previous question)

7.       Figure 4: How many times of experiments have you done? At least we need three repeats. The authors must provide a table to include the strength and ductility error bar.

8.       Figure 5: Why SiC part seems to have more dimples, but AZ91 have more flat surfaces? AZ91 is more brittle than SiC?

9.       Still figure 5: Zoom-in images in the current “SiC” zone (where a lot of curves and wavy surfaces are exposed) should be added.

10.   One thing missing in this paper is the strengthening mechanism discussion and quantification: For compression and toughness, the strengthening mechanism still works. Please refer to and use the equations in ref. [3] to have this discussion.

[3] Thermally stable ultrafine grained copper induced by CrB/CrB2 microparticles with surface nanofeatures via regular casting. G Yao, C Cao, S Pan, J Yuan, I De Rosa, X Li. Journal of Materials Science & Technology 58, 55-62

Author Response

Dear reviewers,

Thank you for your letter and for the reviewers’ comments concerning our manuscript entitled “High Modulus Laminated SiCp/AZ91 Material With Adjustable Microstructure and Mechanical Properties by Adjusting the Densities of the Ceramic Layers” (ID: materials-2559464). Those comments are all valuable and very helpful for revising and improving our paper, as well as the important guiding significance to our researches. We have studied comments carefully and have made correction which we hope meet with approval. Below summarizes the details of the answers to the questions, and revisions made in the manuscript, in which the original comments are written in plain black text, the responses in blue text, and the changes in the manuscript are in red text. We hope that the amendments and responses make the revised manuscript better readable and satisfied with the editor and reviewers. If not, we really appreciate to have the chance to reconsider your additional comments in future communication.

Reviewers' comments:

Reviewer #3

This paper is easy to read, and the logic is clear. But the authors fail to highlight their data in the abstract, and some parts of the main content raised my technical concerns. I recommend a major revision.

Response: Thank you for this positive evaluation. And, the related responses to the comments are provided in the following section, which indeed improve the readability of the paper, and also greatly improve its quality.

1.In the abstract, the authors mention “toughness” with no data support – They should add their toughness number here.

Response 1: I 'm sorry that I meant compressive strain, so I changed toughness to compressive strain, and the specific value of 8.77 % has been given.

2.When it comes to the Mg properties in abstract, they should be specific about the exact numbers for those properties: Exactly how light? How high the specific strength is? Etc.

Response 2: Much thanks for the reviewer’s valuable advice! By comparing the Laminated SiCp/AZ91 Material with the magnesium alloy, the advantages of the composite material can be highlighted. For this reason, we supplemented the compression experiment of the AZ91 alloy, the compressive strength of alloy is 314MPa±12MPa. As shown in Supplementary Fig.1, compared with the AZ91 alloy, the compressive strength of the composite was increased by 100 %. The focus of our research is mainly on the effect of layered configuration regulation on the properties of magnesium matrix composites. So, the paper only put the performance of composite materials. In order to further highlight its impact on composite materials, the line16–21 in page1, has been revised as follows:

“The increased alloy content in the ceramic layer not only inhibits crack initiation but also hinders crack propagation, thereby endowed with excellent compressive strength and compressive strain to the SiCp/AZ91 laminated material. At the sintering temperature of 900°C, the SiCp/AZ91 laminated material exhibits impressive compressive strength and strain values of 623MPa and 8.77%, respectively, which demonstrates an excellent match of strength and toughness. In addition, the change of sintering temperature has a greater impact on the flexural properties of the composites. At 1000℃, the compressive strength and strain of the composites are 400±19.09 MPa and 0.73±0.1 %, respectively. When the temperature is reduced to 800℃, the strength and strain are increased to 533±27 MPa and 1.08±0.09 %, respectively.”

Supplementary Fig. 1 Compressive strength and compressive strain of AZ91 alloy and the laminated SiCp/AZ91 Material

3.The processing involves freeze casting, sintering, and infiltration, which are confusing by pure words (in introduction and methods part). The authors should add a process/procedure illustration plot to show the critical process parameters.

Response 3: Much thanks for the reviewer’s valuable advice! There is indeed a process illustration that is more conducive to understanding the process of the experiment, so add a process illustration to the article(Fig.1). In addition, the reference has been described in detail [10]. So, in the introduction did not do too much description, but its principle is consistent with the Fig. 1.

Figure 1 Flow chart of freeze casting process

4.“crack initiation in composites is primarily attributed to the ceramic layer”: I have doubt for this claim. Indeed, it is the interface of ceramics and metals in composites that contribute to the crack initiation, as there is lattice mismatch. Please learn from Ref. [1-2] and have a more solid discussion.

[1] Geng, J., Li, Y., Xiao, H., Li, H., Sun, H., Chen, D., Wang, M. and Wang, H., 2021. Study fatigue crack initiation in TiB2/Al-Cu-Mg composite by in-situ SEM and X-ray microtomography. International Journal of Fatigue, 142, p.105976.

[2] Nanoparticles enabled mechanism for hot cracking elimination in aluminum alloys. M Sokoluk, J Yuan, S Pan, X Li. Metallurgical and Materials Transactions A 52 (7), 3083-3096

Response 4: Much thanks for the reviewer’s valuable advice! We have deeply studied the literature provided by the reviewers, and combined with the relevant literature [49–58]. Based on the theory in the literature, we have a deep understanding of the fracture behavior of layered materials. For metal layered materials, it is generally composed of soft metals and hard metals. During the preparation of the material, a hard phase will be formed at the interface of the soft and hard metals. Therefore, in the subsequent deformation process, cracks initiate due to the cracking of the hard phase, or the interface debonding between the hard phase and the matrix, and most of the cracks propagate along the layered interface, which will eventually lead to the failure of the metal layered material. For the laminated SiCp/AZ91 Material, the hard layer is SiCp/AZ91 composite, which is composed of the SiCp and the AZ91 alloy, thus it is different from the existing metal layered materials. For the interface between the composite layer and the alloy layer and the interior of the composite layer, the interior of the composite material is more prone to stress concentration. The main reason is that the stress concentration is easy to occur at the interface between the particles and the matrix, and the more the particle content, the greater the stress concentration. Therefore, compared with the alloy layer, the content of particles in the composite material is more, so stress concentration is easy to occur in the particle-intensive area. It is precisely because the stress concentration is easy to occur inside the composite layer, and the alloy layer is relatively soft, which can alleviate the composite layer. Therefore, compared with the matrix, the presence of particles near the interface is prone to stress concentration. Therefore, during the stress process, the interface between the SiC particles and the matrix in the composite layer preferentially generates stress concentration and initiates cracks, rather than the interface between the composite layer and the alloy layer.

5.You used Al2O3 and Y2O3 as sintering aid. How do you confirm they are gone after sintering? If they are still there (as told in your Figure 2), can they react with Mg? Why? Need more explanation.

Response 5: Much thanks for the reviewer’s valuable advice! It is generally believed that the addition of Y2O3 can promote the reaction of Al2O3 and SiO2 to form 3Al2O3·2SiO2 [59] . In addition, Al2O3 and Y2O3 were not detected in the XRD images, but mullite was formed, indicating that Al2O3 and SiO2 reacted to form mullite. However, due to the low content of Y2O3, it was not detected, so we could not confirm that Y2O3 did not exist. In addition, Al2O3 has been reported in reference, Al2O3 reacts with Mg at 950°C [60,61]. And Y2O3 is relatively stable below 1000°C. The temperature of the molten alloy during preparation is 700 °C, because this temperature is lower than the reaction temperature of Al2O3 and Y2O3, so we believe that even if it exists, it will not react with Mg element.

6.Equation 1-5: Need to add the Gibbs free energy change (I think this will also help my previous question)

Response 6: Much thanks for the reviewer’s valuable advice! The Gibbs free energy changes of Equation 1-2 at 25℃ are  and [52]. The reaction temperature of Equation 3-5 in the existing literature is 1500℃, and our temperature is 800℃-1000℃, which is not consistent with literatures [60]. Therefore, in order to describe more rigorously, the Gibbs free energy is not put in the paper, but we are very grateful for this opinion. In the next work, we will analyze the thermodynamics and kinetics of Al2O3 and SiC ceramics in the sintering process, and study the changes of Gibbs free energy at different temperatures.

7.Figure 4: How many times of experiments have you done? At least we need three repeats. The authors must provide a table to include the strength and ductility error bar.

Response 7: Much thanks for the reviewer’s valuable advice! Each compression experiment was repeated 5 times. Figure 4.has been revised as follows:

Figure 5. Longitudinal section of the SiCp/AZ91 laminated material at different sintering temperatures (a) Compressive stress-strain curve (b) compressive stress-strain bar graph(c) Modulus of elasticity

8.Figure 5: Why SiC part seems to have more dimples, but AZ91 have more flat surfaces? AZ91 is more brittle than SiC?

Response 8: Much thanks for the reviewer’s valuable advice! It is interesting that the SiC layer has many dimples and the AZ91 layer with good toughness is relatively smooth. However, it does not mean that AZ91 is more brittle, because the formation of dimples in the SiC layer is different from that in the metal material, which is due to the residual dimples caused by the cracking of the SiCp. Because the composite material is easy to produce stress concentration near the SiC particles when loading. The larger internal stress leads to the preferential cracking near the SiC particles in the ceramic layer, which leads to the formation of holes in the ceramic layer.

9.Still figure 5: Zoom-in images in the current “SiC” zone (where a lot of curves and wavy surfaces are exposed) should be added.

Response 9: Much thanks for the reviewer’s valuable advice! The enlarged view of the SiC layer in the composite was added, the line268–277 in page7-8, has been revised as follows:

“When the composite is loaded along the freezing direction, the ceramic and metal layers experience simultaneous stress, with the metal layer initially deforming upon stress trans-fer at the layer interface [14,30]. According to the enlarged image in the SiC layer, there are more cracks in the SiC layer of the composite, while the AZ91 layer is relatively smooth. This is because the composite is prone to stress concentration near the SiC particles during the loading process. The larger internal stress leads to the preferential initiation of cracks near the SiC particles in the ceramic layer, which leads to the formation of holes in the ceramic layer. The SiCp layer primarily bears the load, generating multi-directional stress within its structure, while the alloy within the layer helps alleviate stress concentration in the SiC particles.”

Figure 6. Typical fracture surfaces after compression at room temperature (a) (d) 800°C (b) (e) 900°C (c) (f) 1000°C.

10.One thing missing in this paper is the strengthening mechanism discussion and quantification: For compression and toughness, the strengthening mechanism still works. Please refer to and use the equations in ref. [3] to have this discussion.

[3] Thermally stable ultrafine grained copper induced by CrB/CrB2 microparticles with surface nanofeatures via regular casting. G Yao, C Cao, S Pan, J Yuan, I De Rosa, X Li. Journal of Materials Science & Technology 58, 55-62

Response 10: Much thanks for the reviewer’s valuable advice! After in-depth study of the literature you provide, it has been greatly inspired, the line232 – 256 in page6-7, has been revised as follows:

“The elastic modulus reaches 105GPa at 1000-L. The Young's modulus enhancement is quite pronounced, especially when considering that AZ91 alloys have a modulus of only 43GPa. This enhancement is primarily attributed to the high Young's modulus of SiC particles (380-470GPa) and their robust bonding with the matrix [27]. The upper limit of Young 's modulus of composites can be calculated by Eq (6) [28]:

Ec=Em×(1-Vp)+Ep×Vp                                                                               (6)

Where Ec, Em, and Ep are the Young 's moduli of the composites, the matrix, and the reinforcing particles, respectively. The upper limit of the predicted Young 's modulus of the laminated SiCp/AZ91 composites is 144-171.1GPa. The obvious experimental value is lower than the theoretical value, which is due to the anisotropy of the composite material, which has a significant effect on its elastic modulus. Therefore, according to the Halpin-Tsai Eq (7-8), the elastic modulus of the longitudinal section of the layered AZ91/SiC composite is further calculated [29]:

 E=EAZ91(1+2sqφ)/(1-qφ)                                                                               (7)

q=(ESiC-EAZ91)/(ESiC+2sEAZ91)                                                                        (8)

Where E, ESiC and EAZ91 are the elastic modulus of composites, SiC ceramics and AZ91 alloy matrix respectively, s is the aspect ratio of reinforced particles, and φ is the volume fraction of reinforced particles. The Young 's modulus of the laminated SiCp/AZ91 composites with a volume fraction of 30 % is calculated to be 77-94GPa when the s value is 1. The longitudinal elastic modulus of 800-L, 900-L and 1000-L are actually 94GPa, 98GPa and 105GPa, respectively, which are slightly higher than the calculated values. This may be related to the fact that the SiC preform is sintered more compact, thereby improving the elastic modulus of the composite. However, although the elastic modulus of the composites at 800°C and 900°C decreases and a certain strength is sacrificed, the plasticity is improved. It can be seen that the mechanical properties and elastic modulus of the composites can be significantly affected by adjusting the bonding of SiC particles in the ceramic layer by sintering temperature.”

Supplementary References

[49] H. Nie WeiChen, HongshengWang, FeiLi, TaotaoChi, ChengzhongLi, Xian Rong, A Coupled EBSD/TEM Study on the Interfacial Structure of Al/Mg/Al Laminates, Journal of Alloys and Compounds: An Interdisciplinary Journal of Materials Science and Solid-State Chemistry and Physics 781, (2019).

[50] K. L. Hwu and B. Derby, Fracture of Metal/Ceramic Laminates—I. Transition from Single to Multiple Cracking, Acta Materialia 47, 529 (1999).

[51] K. L. Hwu and B. Derby, Fracture of Metal/Ceramic Laminates—I. Transition from Single to Multiple Cracking, Acta Materialia 47, 529 (1999).

[52] A. B. Pandey, B. S. Majumdar, and D. B. Miracle, Laminated Particulate-Reinforced Aluminum Composites with Improved Toughness, Acta Materialia 49, 405 (2001).

[53] T. Liu, B. Song, G. Huang, X. Jiang, S. Guo, K. Zheng, and F. Pan, Preparation, Structure and Properties of Mg/Al Laminated Metal Composites Fabricated by Roll-Bonding, a Review, Journal of Magnesium and Alloys 10, 2062 (2022).

[54] Wang, J. Z., Zhai, L., M., Yuan, H., Liu, and C. W., Microstructure, Texture and Mechanical Properties of Al/Al Laminated Composites Fabricated by Hot Rolling, Materials Science & Engineering, A. Structural Materials: Properties, Misrostructure and Processing (2015).

[55] H. Ghorbani and R. Jamaati, Simultaneous Improvement of Strength and Toughness in Al–Zn–Mg–Cu/Pure Al Laminated Composite via Heterogeneous Microstructure, Materials Science and Engineering: A 880, 145362 (2023).

[56] Y. Tang, W. He, B. Jiang, and F. Pan, Influence of Rolling Deformation on Microstructures and Mechanical Properties of Laminated Mg/Zr Composites, Materials Science and Engineering: A 849, 143460 (2022).

[57] Z. Yu, T. Wang, C. Liu, Y. Ma, and W. Liu, Investigation on Microstructure, Mechanical Properties and Fracture Mechanism of Mg/Al Laminated Composites, Materials Science and Engineering: A 848, 143410 (2022).

[58] R. H. Gao, F. Li, P. Da Huo, and W. T. Niu, Evolution Mechanism of Interfacial Morphological Characteristics for Al/Mg/Al Composite Plate Rolled by the Hard Plate, Materials Today Communications 33, 104257 (2022).

[59] S. Ding, Effect of Y2O3 Addition on the Properties of Reaction-Bonded Porous SiC Ceramics, Ceramics International 32, 461 (2006).

[60] A. Shaga, P. Shen, R. F. Guo, and Q. C. Jiang, Effects of Oxide Addition on the Microstructure and Mechanical Properties of Lamellar SiC Scaffolds and Al-Si-Mg/SiC Composites Prepared by Freeze Casting and Pressureless Infiltration, Ceramics International 9653 (2016).

[61] M. I. Pech-Canul, M. Rodríguez-Reyes, M. A. Pech-Canul, and J. C. Rendón-Angeles, Co-Reinforcing Al/SiC Composites with MgAl2O4 Formed In Situ during the Processing by Non-Assisted Infiltration, Metals & Materials International 17, 923 (2011).

Round 2

Reviewer 3 Report

No more comments. It can be accepted